# A Visit to Sheffield's Remarkable Sewage Works

**Published:** 1923-01

**Authors:** 


### A VISIT TO SHEFFIELD'S REMARKABLE SEWAGE WORKS.

IF Mr. John Haworth, the Manager of the Sheffield Sewage Disposal Department at Wincobank, has considerable inroads made upon his time by deputations from other Councils and by other interested observers, he is paying the penalty of being a pioneer and the possessor of a courteous and interesting personality. We were informed by a friendly guide, who directed us to the works by way of an unhallowed football pitch and a railway footbridge, that Wincobank is the Mecca of all pilgrims interested in questions of sewage disposal; and we can well believe it. Mr. Haworth would modestly disclaim the name of pioneer, but it fits him; and the work which is done under his management and as the result of his experiments is something of which the City of Sheffield recognises the value. The experimental work on a large scale, which we shall shortly describe, has so thoroughly proved its superiority over former methods that a new £50,000 contract is in hand, and it is intended as soon as possible to deal with the whole of the sewage of Sheffield by the new method.

If you would know in a sentence what Mr. Haworth poes at present, it is this: He receives 500,000 gallons of sewage every day, mixes it with "bacterial sludge," shakes it about in the air for about half an hour or so, and then pours into the river close by the effluent stream of water completely purified. The really fascinating feature of the arrangement is that the "bacterial sludge" is itself created by the sewage, so that the sewage is purifying itself with no other assistance than that of the atmosphere. It is most interesting, after seeing the sewage waters pouring in a turgid torrent into the plant at one corner, to stroll round to another corner not many yards away and see the wonderfully clear water that there emerges. Shown a specimen in a test glass we did not make the mistake of an earlier visitor who said that the taste was not at all bad—happily with no ill-effects. But let us go a little more into detail, with Mr. Haworth's friendly guidance, avoiding as far as possible technicalities.

The ordinary sewage disposal works, although themselves a product of so recent a period as the closing years of the last century, are a familiar sight to all travellers. It is sufficient with regard to these to say that in the treatment of sewage on land the sewage is brought into contact with organisms present in the soil, and thus is oxidised and purified. This natural action is intensified by the concentration of similar organisms in immense numbers on the surfaces of solid media, such as the slag, stone, slate, &c., composing the contact bed, the percolating filter, and the slate bed. In all these cases the sewage is brought into contact with the organisms in the presence of oxygen of the air taken into solution on the wetted surfaces.

During the year 1912 a series of experiments was initiated at the Sheffield Sewage Disposal Works in order to ascertain what further treatment was necessary to render the effluents from the existing primary contact beds satisfactory to the Rivers Authority. These experiments included treatment of the sewage by aeration.

During the progress of this work a paper was published by Messrs. Ardern and Lockett describing certain experiments carried out in Manchester. Their investigations showed that if a liquid containing organic solids, such as deposits from sewage, was submitted to aeration for a sufficient period, namely, until a high degree of oxidation occurred, the sludge then contained a large number of organisms of varying types and was capable of purifying sewage brought into contact with it in a few hours, provided that air in a finely divided state was continuously blown through the mixture of sludge and sewage. The bacterial sludge thus produced was called " activated sludge."

The great value of these investigations lay in the demonstration that sewage could be purified by bringing it into direct contact with the bacterial sludge, without the necessity of providing solid surfaces, and that contact beds and percolating filters could thus be dispensed with, and the operations of sewage disposal be reduced to treatment in tanks.

In view of this development some of the experimental tanks already in use at Sheffield were brought into requisition for further experiment in aeration by blowing, but considerable difficulty was encountered with regard to the blowing of air into the sewage through small orifices.

Further experiments were then devised having chiefly in view the following, among other, considerations :—(1) That the air blown into the sewage performs two functions, viz., (*a*) the supply of oxygen in solution to maintain the biological life, and (*b*) the agitation of the sludge with the liquid, the greater proportion of the air being used for the latter purpose. (2) That these functions might possibly be separated : the agitation might be accomplished by direct mechanical appliances and the smaller amount of air in solution be supplied by other means. These experiments proved successful.

Apart from the gauging chamber and motor-house, the whole installation now consists of (1) screens and detritus or grit tanks, (2) aeration tanks, (3) retention chambers, and (4) settling tanks.

The necessary supply of oxygen in solution for maintaining the organisms created in the sewage is obtained by the constant change of surface exposed to the operation of the atmosphere. The aeration tanks, which are of concrete, can be compared in appearance to a vast grid-iron. The sewage traverses the whole internal measurement of the grid, up and down, agitated anew at the end of each double journey by paddle wheels.

A further surface change is produced by the narrowing of the channel where the liquid turns the corners, as this causes an additional swirling and eddying surface motion. The bacterial sludge is already awaiting in the tanks the arrival of the sewage and mixes with it as it rushes through the channels of the aeration chamber.

The liquid passes from the aeration tank to the retention chambers. These are triangular in section ; the wall adjoining the aeration tank is vertical, and the liquid enters the chambers by passing over it. The opposite wall is sloping, and at the top of this is formed the weir over which the liquid discharges into the feed channel to the settling tanks. In the bottom of the vertical wall are openings through which any sludge settling in the retention chambers may return to the aeration tank. The object of the retention chambers is, by the greatly diminished velocity of flow obtained in them, to secure partial settlement of the liquid before it passes to the settling tanks, and so " retain " as much sludge as possible in the aeration tank. This advantage is threefold ; it reduces the amount of sludge to be pumped, less sludge reaches the settling tanks, and thereby their settling capacity is increased, and there is less sludge temporarily out of action.

The four sides of each settling tank form the weir over which the effluent passes to the collecting channels, or direct to the gauging chamber, where the flow is registered. From the chamber the effluent empties into the discharge culvert to the river.

The sludge from the settling tanks is now ready to be pumped back into the aeration tanks to carry on the good work, and so the circle is complete. About one per cent. of the sludge is finally removed each day, that being the equivalent of the new sludge which is created by the incoming sewage. The problem of the most economical way of disposing of the sludge so removed is still under consideration, but it has a very great manurial value, and is free from any smell. It should therefore not be difficult to find a good market for it.

This, in brief outline, is the sewage scheme now in operation at Wincobank. Amongst the deputations visiting the works has been one from Paris, where the authorities are now conducting experiments. Mr. Haworth has been to the Hague to advise the Dutch Authorities with regard to the installation of a similar plant. He has no patent rights in his plant ; he does not consider that finality has been reached—he is not that sort of man. The capital outlay of the plant is less than one-half of that of old filter beds of an equivalent capacity ; the area occupied is one-sixth ; the labour required is merely the time of one man in and about the motor-house. The present plant was only completed at the end of 1920, and already other Councils, having seen the excellence of the results obtained, are following Sheffield's example. The extension of this scheme to other areas up and down the country is unquestionable. All students of health questions will be closely interested in further developments of this valuable contribution to the preservation of the public health.

